# Scaling tree-based automated machine learning to biomedical big data with a feature set selector

**DOI:** 10.1093/bioinformatics/btz470

**Published:** 2019-06-04

**Authors:** Trang T Le, Weixuan Fu, Jason H Moore

**Affiliations:** Department of Biostatistics, Epidemiology and Informatics, Institute for Biomedical Informatics, University of Pennsylvania, Philadelphia, PA 19104, USA

## Abstract

**Motivation:**

Automated machine learning (AutoML) systems are helpful data science assistants designed to scan data for novel features, select appropriate supervised learning models and optimize their parameters. For this purpose, Tree-based Pipeline Optimization Tool (TPOT) was developed using strongly typed genetic programing (GP) to recommend an optimized analysis pipeline for the data scientist’s prediction problem. However, like other AutoML systems, TPOT may reach computational resource limits when working on big data such as whole-genome expression data.

**Results:**

We introduce two new features implemented in TPOT that helps increase the system’s scalability: Feature Set Selector (FSS) and Template. FSS provides the option to specify subsets of the features as separate datasets, assuming the signals come from one or more of these specific data subsets. FSS increases TPOT’s efficiency in application on big data by slicing the entire dataset into smaller sets of features and allowing GP to select the best subset in the final pipeline. Template enforces type constraints with strongly typed GP and enables the incorporation of FSS at the beginning of each pipeline. Consequently, FSS and Template help reduce TPOT computation time and may provide more interpretable results. Our simulations show TPOT-FSS significantly outperforms a tuned XGBoost model and standard TPOT implementation. We apply TPOT-FSS to real RNA-Seq data from a study of major depressive disorder. Independent of the previous study that identified significant association with depression severity of two modules, TPOT-FSS corroborates that one of the modules is largely predictive of the clinical diagnosis of each individual.

**Availability and implementation:**

Detailed simulation and analysis code needed to reproduce the results in this study is available at https://github.com/lelaboratoire/tpot-fss. Implementation of the new TPOT operators is available at https://github.com/EpistasisLab/tpot.

**Supplementary information:**

Supplementary data are available at *Bioinformatics* online.

## 1 Introduction

For many bioinformatics problems of classifying individuals into clinical categories from high-dimensional biological data, performance of a machine learning (ML) model depends greatly on the problem it is applied to (Olson *et al.*, 2017, 2018). In addition, choosing a classifier is merely one step of the arduous process that leads to predictions. To detect patterns among features (e.g. clinical variables) and their associations with the outcome (e.g. clinical diagnosis), a data scientist typically has to design and test different complex ML frameworks that consist of data exploration, feature engineering, model selection and prediction. Automated machine learning (AutoML) systems were developed to automate this challenging and time-consuming process. These intelligent systems increase the accessibility and scalability of various ML applications by efficiently solving an optimization problem to discover pipelines that yield satisfactory outcomes, such as prediction accuracy. Consequently, AutoML allows data scientists to focus their effort in applying their expertise in other important research components such as developing meaningful hypotheses or communicating the results.

Grid search, random search (Bergstra and Bengio, 2012), Bayesian optimization (Brochu *et al.*, 2010) and evolutionary algorithm (EA) (Eiben and Smith, 2010) are four common approaches to build AutoML systems for diverse applications. Both grid search and random search could be too computational expensive and impractical to explore all possible combinations of the hyperparameters on a model with high-dimensional search space, e.g. with more than 10 hyperparameters (Dewancker *et al.*, 2016). Bayesian optimization is implemented in both auto-sklearn (Feurer *et al.*, 2015) and Auto-WEKA (Thornton *et al.*, 2013; Kotthoff *et al.*, 2017) for model selection and hyperparameter optimization. Although both systems allow simple ML pipelines including data pre-processing, feature engineering and single model prediction, they cannot build more complex pipelines or stacked models which are necessary for complicated prediction problems. On the other hand, EA can generate highly extensible and complex ML pipelines and ensemble models for data scientists. For example, Recipe (de Sá *et al.*, 2017) uses grammar-based EA to build and optimize ML pipelines based on a fully configurable grammar. Autostacker (Chen *et al.*, 2018) uses basic EA to look for flexible combinations of many ML algorithms that yield better performance. DEvol (https://github.com/joeddav/devol) was designed specifically for deep neural networks and can optimize complex model architecture by using EA to tune hyperparameters related to convolutional/dense layers and optimizer. More recently released, GAMA (Gijsbers and Vanschoren, 2019) performs automatic ensemble of best ML pipelines evaluated by asynchronous EA instead of simply using a single best pipeline for prediction. Progressively, EA enhances AutoML systems with high flexibility in building pipelines in a large search space of ML algorithms and their hyperparameters.

Tree-based Pipeline Optimization Tool (TPOT) is a EA-based AutoML system that uses genetic programing (GP) (Banzhaf *et al.*, 1998) to optimize a series of feature selectors, pre-processors and ML models with the objective of maximizing classification accuracy. Although most AutoML systems primarily focus on model selection and hyperparameter optimization, TPOT also pays attention to feature selection and feature engineering by evaluating the complete pipelines based on their cross-validated score such as mean squared error or balanced accuracy. Given no a priori knowledge about the problem, TPOT has been shown to frequently outperform standard ML analyses (Olson *et al.*, 2016; Olson and Moore, 2016). Effort has been made to specialize TPOT for human genetics research, resulting in a useful extended version of TPOT, TPOT–MDR that features Multifactor Dimensionality Reduction and an Expert Knowledge Filter (Sohn *et al.*, 2017). However, at the current stage, TPOT still requires great computational expense to analyze large datasets such as in genome-wide association studies (GWASs) or gene expression analyses. Consequently, the application of TPOT on real-world datasets has been limited to small sets of features (Le *et al.*, 2018a).

In this work, we introduce two new features implemented in TPOT that helps increase the system’s scalability. First, the Feature Set Selector (FSS) allows the users to pass specific subsets of the features, reducing the computational expense of TPOT at the beginning of each pipeline to only evaluate on a smaller subset of data rather than the entire dataset. Consequently, FSS increases TPOT’s efficiency in application on large datasets by slicing the data into smaller sets of features (e.g. genes) and allowing a genetic algorithm to select the best subset in the final pipeline. Second, Template enables the option for strongly typed GP, a method to enforce type constraints in GP. By letting users specify a desired structure of the resulting ML pipeline, Template helps reduce TPOT computation time and potentially provide more interpretable results.

## 2 Materials and methods

We begin with descriptions of the two novel additions to TPOT, FSS and Template. Then, we provide detail of a real-world RNA-Seq dataset and describe a simulation approach to generate data comparable to the RNA-Seq data. Finally, we discuss other methods and performance metrics for comparison. Detailed simulation and analysis code needed to reproduce the results has been made available on the GitHub repository https://github.com/lelaboratoire/tpot-fss.

### 2.1. Tree-based pipeline optimization tool

TPOT automates the laborious process of designing a ML pipeline by representing pipelines as binary expression trees with ML operators as primitives. Pipeline elements include algorithms from the extensive library of scikit-learn (Pedregosa *et al.*, 2011) as well as other efficient implementations such as extreme gradient boosting. Applying GP with the NSGA-II Pareto optimization (Deb *et al.*, 2002), TPOT optimizes the accuracy achieved by the pipeline while accounting for its complexity. Specifically, to automatically generate and optimize these machine-learning pipelines, TPOT utilizes the Python package DEAP (Fortin *et al.*, 2012) to implement the GP algorithm. Implementation details can be found at TPOT’s active Github repository https://github.com/EpistasisLab/tpot.

#### 2.1.1 Feature set selector

TPOT’s current operators include sets of feature pre-processors, feature transformers, feature selection techniques and supervised classifiers and regressions. In this study, we introduce a new operator called FSS that enables biologically guided group-level feature selection. From pre-defined subsets of features, the FSS operator allows TPOT to select the best subset that maximizes average accuracy in *k*-fold cross validation (5-fold by default). Specifically, taking place at the very first stage of the pipeline, FSS passes only a specific subset of the features onwards, effectively slicing the large original dataset into smaller ones. Hence, with FSS, users can specify subsets of features of interest to reduce the feature space’s dimension at pipeline initialization.

For example, in a gene expression analysis of major depressive disorder (MDD), a neuroscientist can specify collections of genes in pathways of interest and identify the important collection that helps predict the depression severity. Similarly, in a GWAS of breast cancer, an analyst may assign variants in the data to different subsets of potentially related variants and detect the subset associated with the breast cancer diagnosis. In general, the FSS operator takes advantage of previous compartmentalization of the feature space to smaller subsets based on a priori expert knowledge about the biomedical dataset. From here, TPOT learns and selects the most relevant group of features for outcome prediction. When compared with TPOT’s existing Selector operators, FSS selects features at the group level instead of individual level.

#### 2.1.2 Template

Parallel with the establishment of the FSS operator, we now offer TPOT users the option to define a Template that provides a way to specify a desired structure for the resulting ML pipeline, which will reduce TPOT computation time and potentially provide more interpretable results.

**Fig. 1.**
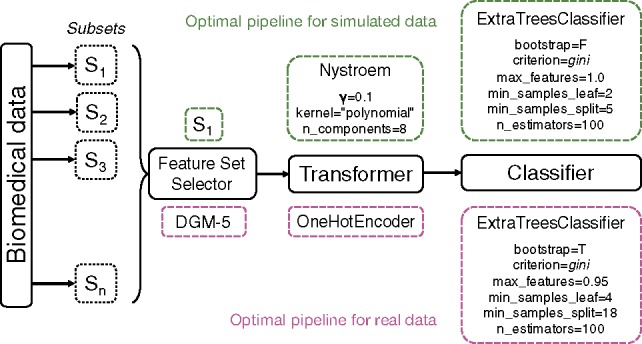
Template’s general workflow with TPOT-FSS and example pipelines. Final pipelines with optimized parameters are shown for simulated data (top, green) and real-world RNA-Seq data (bottom, mauve). The specific operators selected in optimal pipelines include built-in TPOT’s operators (OneHotEncoder, FeatureSetSelector) and functions from the library of scikit-learn (ExtraTreesClassifier, Nystroem)

Current implementation of Template supports linear pipelines, or path graphs, which are trees with two nodes (operators) of vertex degree 1, and the other *n* − 2 nodes of vertex degree 2. Further, Template takes advantage of the strongly typed GP framework that enforces data-type constraints (Montana, 1995) and imposes type-based restrictions on which element (i.e. operator) type can be chosen at each node. In strongly typed GP, while the fitness function and parameters remain the same, the initialization procedure and genetic operators (e.g. mutation, crossover) must respect the enhanced legality constraints (Montana, 1995). With a Template defined, each node in the tree pipeline is assigned one of the five major operator types: FSS, feature selector, feature transformer, classifier or regressor. Moreover, besides the major operator types, each node can also be assigned more specifically as a method of an operator, such as decision trees for classifier. An example Template is FSS → Feature transform → Decision trees (Fig. 1).

### 2.2 Datasets

We apply TPOT with the new FSS operator on both simulated datasets and a real world RNA expression dataset. With both real-world and simulated data, we hope to acquire a comprehensive view of the strengths and limitations of TPOT in the next generation sequencing domain.

#### 2.2.1 Simulation methods

The simulated datasets were generated using the R package privateEC, which was designed to simulate realistic effects to be expected in gene expression or resting-state fMRI data. In this study, to be consistent with the real RNA-Seq dataset (described below), we simulate interaction effect data with *m *=* *200 individuals (100 cases and 100 controls) and *P *=* *5000 real-valued features with 4% functional (true positive association with outcome) for each training and testing set. Full details of the simulation approach can be found in Lareau *et al.* (2015) and Le *et al.* (2018b). Briefly, the privateEC simulation induces a differential co-expression network of random normal expression levels and permutes the values of targeted features within the cases to generate interactions. Further, by imposing a large number of background features (no association with outcome), we seek to assess TPOT–FSS’s performance in accommodating large numbers of non-predictive features.

To closely resemble the module size distribution in the RNA-Seq data, we first fit a Γ distribution to the observed module sizes then sample from this distribution values for the simulated subset size, before the total number of features reaches 4800 (number of background features). Then, the background features were randomly placed in each subset corresponding to its size. Also, for each subset $S_{i},\;i\;=\;1,\ldots ,\;n$, a functional feature $s_{j}$ belongs to the subset with the probability

(1) $$P(s_{j}\in S_{i})∼1.618^{-i}$$

where 1.618 is an approximation of the golden ratio and yields a reasonable distribution of the functional features: they are more likely to be included in the earlier subsets (Subsets 1 and 2) than the later ones.

#### 2.2.2 Real-world RNA-Seq expression data

We employ TPOT-FSS on an RNA-Seq expression dataset of 78 individuals with MDD and 79 healthy controls (HCs) from Le *et al.* (2018b). RNA expression levels were quantified from reads of 19 968 annotated protein-coding genes and underwent a series of pre-processing steps including low read-count and outlier removal, technical and batch effect adjustment, and coefficient of variation filtering. Consequently, whole blood RNA-Seq measurements of 5912 transcripts were used to identify depression gene modules (DGMs) based on a read alignment protocol that enriched for the expression of antisense RNA. In this study, we apply TPOT-FSS to this processed dataset to verify our method’s ability to select the subset of features that is important for predicting the MDD outcome. The primary antisense-enriched dataset, along with a second pre-processed dataset enriched for gene expression, is available on the Github repository of the original MDD study (https://github.com/insilico/DepressionGeneModules). We use the interconnected genes in 23 DGMs identified from the original RNA-Seq gene network module analysis (Le *et al.*, 2018b) as input for the FSS operator. We remark that these modules were constructed by an unsupervised ML method with dynamic tree cutting from a co-expression network. As a result, this prior knowledge of the gene structure does not depend on the diagnostic phenotype and thus yields no bias in the downstream analysis of TPOT-FSS.

### 2.3 Performance assessment

For each simulated and real-world dataset, after randomly splitting the entire data in two balanced smaller sets (75% training and 25% holdout), we trained TPOT-FSS with the Template FeatureSetSelector-Transformer-Classifier on training data to predict class (e.g. diagnostic phenotype in real-world data) in the holdout set. We assess the performance of TPOT-FSS by quantifying its ability to correctly select the most important subset (containing most functional features) in 100 replicates of TPOT runs on simulated data with known underlying truth. To prevent potential overfitting, we select the pipeline that is closest to the 90th percentile of the cross-validation accuracy to be optimal. This rationale is motivated by a similar procedure for optimizing the penalty coefficient in regularized regression where the most parsimonious model within one standard error of the minimum cross-validation error is picked (Hastie *et al.*, 2009). We compare the holdout (out-of-sample) accuracy of TPOT-FSS’s optimal pipeline on the holdout set with that of standard TPOT (with Transformer-Classifier Template, no FSS operator) and eXtreme Gradient Boosting (Chen and Guestrin, 2016), or XGBoost, which is a fast and an efficient implementation of the gradient tree boosting method that has shown much utility in many winning Kaggle solutions (https://www.kaggle.com/) and been successfully incorporated in several neural network architectures (Ren *et al.*, 2017; Zheng *et al.*, 2017). In the family of gradient boosted decision trees, XGBoost accounts for complex non-linear interaction structure among features and leverages gradient descents and boosting (sequential ensemble of weak classifiers) to effectively produce a strong prediction model. To obtain the optimal performance for this baseline model, we tune XGBoost hyperparameters using TPOT Template with only one classifier XGBClassifier, which is imported from the xgboost python package. Because of stochasticity in the optimal pipeline from TPOT-FSS, standard TPOT and the tuned XGBoost model, we fit these models on the training data 100 times and compare 100 holdout accuracy values from each method. We choose accuracy to be the metric for comparison because phenotype is balanced in both simulated data and real-world data.

### 2.4 Article drafting

This article is collaboratively written using Manubot (Himmelstein *et al.*, 2019), a software that supports open paper writing via GitHub using the Markdown language. Manubot uses continuous integration to monitor changes and automatically update the article. Consequently, the latest version of this article is always available at https://trang1618.github.io/tpot-fss-ms/.

## 3 Results

Our main goal is to test the performance of methods to identify features that discriminate between groups and optimize the classification accuracy.

### 3.1 TPOT-FSS recommends optimal pipelines

As discussed earlier in Section 2, the optimal pipeline from TPOT–FSS and standard TPOT is selected to be closest to the 90th percentile of the cross-validation accuracy. The optimal model of XGBoost holds properly tuned hyperparameters. For simulated dataset, the optimal pipeline selects subset $S_{1}$ then constructs an approximate feature map for a linear kernel with Nystroem, which uses a subset of the data as the basis for the approximation. The final prediction is made with an extra-trees classifier that fits a number of randomized decision trees on various sub-samples of the dataset with the presented optimized parameters (Fig. 1). For the real-world dataset, the most optimal pipeline selects subset DGM-5, one-hot encode the features, then, similar to simulated data, makes the final prediction with an extra-trees classifier with a different set of optimized parameters (Fig. 1).

### 3.2 Accuracy assessment of optimal pipelines

We compare the accuracy produced by optimal models from TPOT–FSS, standard TPOT and XGBoost on classifying a simulated dataset with moderate interaction effect. We assign values of the effect size in the simulations to generate adequately challenging datasets so that the methods’ accuracies stay moderate and do not cluster around 0.5 or 1. The resulting accuracy values are comparable to those in real-world data. The dataset is split into 75% training and 25% holdout. The three models are built from the training dataset; then, the trained model is applied to the independent holdout data to obtain the holdout accuracy.

We also apply the three methods to the RNA-Seq study of 78 MDD subjects and 79 HCs described in (Le *et al.*, 2018b). The dataset contains 5912 genes after pre-processing and filtering (see Section 2 for more detail). We excluded 277 genes that did not belong to 23 subsets of interconnected genes (DGMs) so that the dataset remains the same across the three methods. As with simulated data, all models are built from the training dataset (61 HC and 56 MDD), then the trained model is applied to the independent holdout data (18 HC and 22 MDD).

**Fig. 2.**
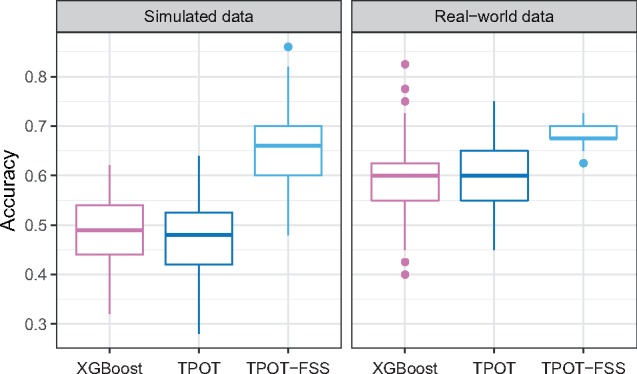
Performance comparison of three models: tuned XGBoost, optimal pipeline from standard TPOT and optimal pipeline from TPOT-FSS. In both simulated and real-world expression datasets, TPOT-FSS optimal pipelines significantly outperform those of XGBoost and standard TPOT

For the simulated data, across all 100 model fits, the optimal TPOT-FSS pipeline yields an average holdout prediction accuracy of 0.65, while the standard TPOT without FSS and tuned XGBoost models respectively report an average holdout accuracy of 0.48 and 0.49 (Fig. 2). This overfitting in the performance of these other two models is likely due to the models’ high flexibility that *over-learns* the training data, especially with the presence of many noisy background features.

Meanwhile, for the real-world RNA-Seq data, the optimal TPOT-FSS pipeline yields an average holdout prediction accuracy of 0.68, while the standard TPOT without FSS and tuned XGBoost models produce average holdout accuracies of 0.60 and 0.59, respectively across all 100 model fits (Fig. 2). In summary, the optimal models from standard TPOT and XGBoost perform better in real-world data compared with simulated data but still worse than that of TPOT-FSS. In both datasets, separate Welch two-sample one-sided *t*-tests show TPOT-FSS optimal pipelines significantly outperform those of XGBoost and standard TPOT (all *P* values $<10^{-15}$).

### 3.3 Consistency in selecting subsets of TPOT-FSS

**Fig. 3.**
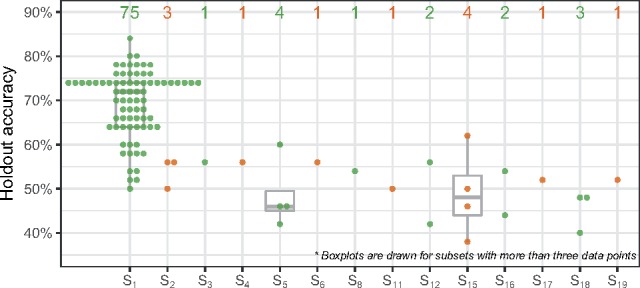
TPOT-FSS’s holdout accuracy (vertical) with selected subset (horizontal) in 100 replications on the simulated data. Number of pipeline inclusions of each subset is displayed above the boxplots. Subset *S*_1_ is the most frequent to be included in the final pipeline and yields the best prediction accuracy in the holdout set. Alternating colors separate adjacent subsets for better visualization

Our simulation design produces a reasonable distribution of the functional features in all subsets, of which proportions are shown in Supplementary Table S1. According to Equation (1), the earlier the subset, the more functional features it has. Therefore, our aim is to determine how well TPOT-FSS can identify the first subset ($S_{1}$) that contains the largest number of informative features. In 100 replications, TPOT-FSS correctly selects subset $S_{1}$ in 75 resulting pipelines (Fig. 3), with the highest average holdout accuracy (0.69 across all 75 pipelines).

**Fig. 4.**
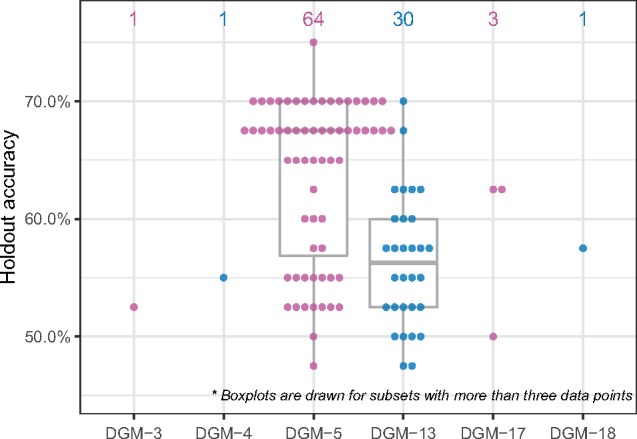
TPOT-FSS’s holdout accuracy (vertical) with selected subset (horizontal) in 100 replications on the RNA-Seq data. Number of pipeline inclusions of each subset is displayed above the boxplots. Subsets DGM-5 and DGM-13 are the most frequent to be included in the final pipeline. Pipelines that include DGM-5, on average, produce higher MDD prediction accuracies in the holdout set

**Fig. 5.**
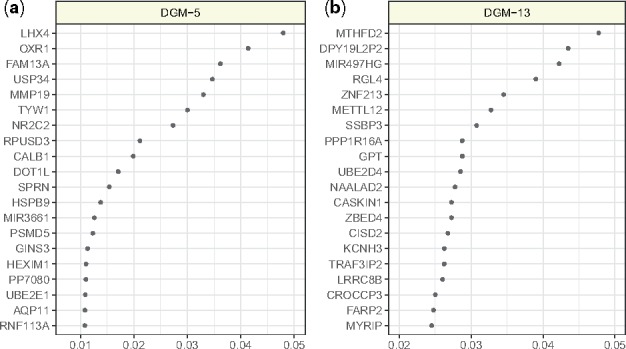
Permutation importance scores of the top twenty features in the optimal pipeline that selects (**a**) DGM-5 and one that selects (**b**) DGM-13. Comprehensive importance scores of the all features computed by permutation from the optimal pipelines are provided in Supplementary Table S2

For the RNA-Seq data, in 100 replications, TPOT-FSS selects DGM-5 (291 genes) 64 times to be the subset most predictive of the diagnosis status (Fig. 4), with the highest average holdout accuracy of 0.636 across 64 pipelines. In the previous study with a modular network approach, we showed that DGM-5 has statistically significant associations with depression severity measured by the Montgomery-Åsberg Depression Scale (MADRS). Although there is no direct link between the top genes of the module (Fig. 5a) and MDD in the literature, many of these genes interact with other MDD-related genes. For example, NR2C2 interacts with FKBP5 gene whose association with MDD has been strongly suggested (Binder *et al.*, 2004; Lavebratt *et al.*, 2010; Tatro *et al.*, 2009). Many of DGM-5’s top genes, including FAM13A, NR2C2, PP7080 and OXR1, were previously shown to have significant association with the diagnosis phenotype using a Relief-based feature selection method (Le *et al.*, 2019). Further, with 82% overlap of DGM-5’s genes in a separate dataset from the RNA-Seq study by Mostafavi *et al.* (2013), this gene collection’s enrichment score was also shown to be significantly associated with the diagnosis status in this independent dataset.

After DGM-5, DGM-13 (134 genes) was selected by TPOT-FSS 30 times (Fig. 4), with an average holdout accuracy of 0.563 across 30 pipelines. The previous network approach did not find statistically significant association between this module’s enrichment score and the MADRS. Although many of the top genes (Fig. 5b) do not have direct disease association, several have been linked to depression-like behavior in animal studies such as PPP1R16A (Sibille *et al.*, 2009) and CASKIN1 (Katano *et al.*, 2018). The RGL4 gene, a Ral guanine nucleotide dissociation stimulator, was found to have a rare protein disruptive variant in at least one suicide patient among 60 other mutations (Tombácz *et al.*, 2017).

### 3.4 Computational expense

For a dataset of the size simulated in our study (*m *=* *200 samples and *P *=* *5000 attributes), standard TPOT has a 18.5-h runtime on a low performance computing machine with an Intel Xeon E5-2690 2.60 GHz CPU, 28 cores and 256 GB of RAM, whereas TPOT-FSS has a 65-min runtime, ∼17 times faster. On the same low performance computing machine, each replication of standard TPOT on the RNA-Seq data takes on average 13.3 h, whereas TPOT-FSS takes 40 min, ∼20 times faster.

## 4 Discussion

To our knowledge, TPOT-FSS is the first AutoML tool to offer the option of feature selection at the group level. Previously, it was computationally expensive for any AutoML program to process biomedical big data. TPOT-FSS is able to identify the most meaningful group of features to include in the prediction pipeline. We assess TPOT-FSS’s holdout prediction accuracy compared with standard TPOT and XGBoost, another state-of-the-art ML method. We apply TPOT-FSS to real-world RNA-Seq data to demonstrate the identification of biologically relevant groups of genes.

Implemented with a strongly typed GP, Template provides more flexibility by allowing users to pre-specify a particular pipeline structure based on their knowledge, which speeds up AutoML process and provides potentially more interpretable results. For example, in high-dimensional data, dimensionality reduction or feature selection algorithms are preferably included at the beginning of the pipelines via Template to identify important features and, meanwhile, reduce computation time. For datasets with categorical features, pre-processing operators for encoding those features such as one-hot encoder should be specified in the pipeline structure to improve pipelines’ performance. Template was utilized in this study to specify the FSS as the first step of the pipeline, which enables the comparison between the two TPOT implementations, with and without FSS.

We simulated data of the similar scale and challenging enough for the models to have similar predictive power as in the real-world RNA-Seq data. TPOT-FSS correctly selects the subset with the most important features in the majority of replications and produces high average holdout accuracy of 0.69. In both simulated and RNASeq data, the final TPOT-FSS pipeline outperforms that of standard TPOT and XGBoost. The low holdout accuracies of standard TPOT and XGBoost are expected because of the few signals in a high-dimenional feature space of the data. Meanwhile, TPOT-FSS finds a more compact feature space to operate on, resulting in higher prediction accuracy and lower computational expense.

Interestingly enough, TPOT-FSS repeatedly selects DGM-5 to include in the final pipeline. In a previous study, we showed DGM-5 and DGM-17 enrichment scores were significantly associated with depression severity (Le *et al.*, 2018b). We also remarked that DGM-5 contains many genes that are biologically relevant or previously associated with mood disorders (Le *et al.*, 2018b) and its enriched pathways such as apoptosis indicates a genetic signature of MDD pertaining to shrinkage of brain region-specific volume due to cell loss (Eilat *et al.*, 1999; McKinnon *et al.*, 2009). TPOT-FSS also selects DGM-13 as a potentially predictive group of features with smaller average holdout accuracy compared with DGM-5 (0.563 < 0.636). The lack of previously found association of these genes with the phenotype is likely because MDD is a complex disorder of heterogeneous etiology (Levinson *et al.*, 2014). Hence, the clinical diagnosis is the accumulative result of coordinated variation of many genes in the module, especially ones with high importance scores. Future studies to refine and characterize genes in DGM-13 as well as DGM-5 may deploy expression quantitative trait loci (e-QTL) or interaction QTL analysis to discover disease-associated variants (Lareau *et al.*, 2016).

Complexity–interpretability tradeoff is an important topic to discuss in the context of AutoML. Although arbitrarily shaped pipelines may yield predictions competitive to human-level performance, these pipelines are often too complex to be interpretable. Vice versa, a simpler pipeline with defined steps of operators may be easier to interpret but yield suboptimal prediction accuracy. Finding the balance between pipeline complexity, model interpretation and generalization remains a challenging task for AutoML application in biomedical big data. With FSS, in the terminology of EA, each pipeline individual of a TPOT generation during optimization holds lower complexity due to the selected subset’s lower dimension compared with that of the entire dataset. We hope that, with the complexity reduction from imposing a strongly type GP template and FSS, a small loss in dataset-specific predictive accuracy can be compensated by considerable increase in interpretability and generalizability. In this study, the resulting TPOT-FSS pipelines are more interpretable with only two simple optimized operators after the FSS: a transformer and a classifier. In the case of the expression analysis, these pipelines also highlight two small sets of interconnected genes that contain candidates for MDD and related disorders. Additionally, complexity reduction results in more efficient computation, which is strongly desirable in biomedical big data analysis.

A limitation of the FSS analysis is the required pre-definition of subsets prior to executing TPOT-FSS. Although this characteristic of an intelligent system is desirable when a prior knowledge on the biomedical data is available, it might pose as a challenge when this knowledge is inadequate, such as when analyzing data of a brand-new disease. Nevertheless, one can perform a clustering method such as *k*-means to group features prior to performing TPOT-FSS on the data. Another limitation of the current implementation of TPOT-FSS is its restricted ability to select only one subset. A future design to support tree structures for Template will enable TPOT-FSS to identify more than one subset that have high predictive power of the outcome. A new operator that combines the data subsets will prove useful in this design. Extensions of TPOT-FSS will also involve overlapping subsets, which will require pipeline complexity reformulation beyond the total number of operators included in a pipeline. Specifically, in the case of overlapping subsets, the number of features in the selected subset(s) is expected to be an element of the complexity calculation. Extension of TPOT-FSS to GWAS is straightforward. However, because of the low predictive power of variants in current GWAS, alternative metrics beside accuracy, balanced accuracy or area under the receiving operator characteristic curve will need to be designed and included in the fitness function of TPOT’s EA.

In this study, we developed two new operators for TPOT, FSS and Template, to enhance its performance on high-dimensional data by simplifying the pipeline structure and reducing the computational expense. FSS helps users leverage domain knowledge to narrow down important features for further interpretation, and Template largely increases flexibility of TPOT via customizing pipeline structure. Future extension and integration of these two operators have the potential to enrich the application of AutoML on different real world biomedical problems.

## Funding

This work was supported by the National Institutes of Health grant numbers LM010098, LM012601 and AI116794. 

*Conflict of Interest*: none declared.

## Supplementary Material

btz470_Supplementary_Files
